# Do skeletal cephalometric characteristics correlate with condylar volume, surface and shape? A 3D analysis

**DOI:** 10.1186/1746-160X-8-15

**Published:** 2012-05-15

**Authors:** Matteo Saccucci, Antonella Polimeni, Felice Festa, Simona Tecco

**Affiliations:** 1 Department of Oral Science, Sapienza University of Rome, Rome, Italy; 2 Department of Oral Science, nano and biotechnology, University G. D’Annunzio, Chieti/Pescara, Chieti, Italy

**Keywords:** Condylar volume, Facial morphology, 3D, Cone beam

## Abstract

**Objective:**

The purpose of this study was to determine the condylar volume in subjects with different mandibular divergence and skeletal class using cone-beam computed tomography (CBCT) and analysis software.

**Materials and methods:**

For 94 patients (46 females and 48 males; mean age 24.3 ± 6.5 years), resultant rendering reconstructions of the left and right temporal mandibular joints (TMJs) were obtained.

Subjects were then classified on the base of ANB angle the GoGn-SN angle in three classes (I, II, III) . The data of the different classes were compared.

**Results:**

No significant difference was observed in the whole sample between the right and the left sides in condylar volume.

The analysis of mean volume among low, normal and high mandibular plane angles revealed a significantly higher volume and surface in low angle subjects (p < 0.01) compared to the other groups.

Class III subjects also tended to show a higher condylar volume and surface than class I and class II subjects, although the difference was not significant.

**Conclusions:**

Higher condylar volume was a common characteristic of low angle subjects compared to normal and high mandibular plane angle subjects. Skeletal class also appears to be associated to condylar volume and surface.

## Introduction

The shape and volume of the condyle in young adults is considered to play an important role in the stability of long-term orthodontic and orthognathic therapies [1]‐ [7].

Since the mandibular condyle undergoes a remodelling process as it responds to continuous stimuli from childhood to adulthood, it is the primary centre of growth in the mandible, where its final dimension of shape and volume could be linked to the relation between the maxillary and mandibular bases.

Being a part of the TMJ structure, the condyle shows a continuous adaptability to functional stimuli. During adulthood, the condyle is often subjected to an ongoing remodelling processes, such as flattening, erosion, sclerosis, osteophytes, and resorption, which could affect its volume and shape [3].

Such changes in the condyle are more associated with a number of clinical conditions: (i) arthritis, which can affect the condylar volume; (ii) the asymmetry, as recently assessed in humans [8]; (iii) anterior disc displacement, as demonstrated in rabbit joints, [9] which have been shown to cause an increase in condylar volume probably because of hyperplasia of condylar cartilage; (iv) an increase in misarticulating surface of the condylar head.

To our knowledge, data on the optimum size or volume of the mandibular condyle are not present in the literature, but they could be indicative and predictive of a precise clinical situation. Therefore, a list of standards, or optimum measurements for size and volume of the mandibular condyle could be useful in predicting risk factors for some pathologies, such as disc displacement.

The cone-beam computed tomography (CBCT) can provide high-resolution images (i.e. with an isotropic resolution ranging from 0.4 to 0.125 mm), short scanning times (10 – 70 seconds), and reduced radiation dose (reportedly up to 15 times lower than that of medical CT scans [1].

CBCT therefore provides the opportunity for Multiplan imaging and three-dimensional (3D) information, that can be useful in the study of condylar morphology.

The purpose of this study was to determine the mandibular condylar volume and surface in a group of adult subjects clinically asymptomatic for TMJ pain and dysfunction [10] using CBCT. The analysis of the condylar volume and surface was performed using custom-made software.

## Material and methods

### The sample

The 3D scans of 188 temporo-mandibular joints (TMJ) of 94 Caucasian adult (mean age 24.3 + 6.5 years, 46 females and 48 males) was retrospectively examined. The subjects did not show pain or dysfunction at TMJ [10] and condylar morphology appeared reasonably normal as well.

All the subjects had taken cone beam evaluation of the stomatognatic apparatus (including the TMJ area) for the following reasons: (i) teeth extraction, such as wisdom teeth; (ii) orthodontic evaluation of unerupted teeth; (iii) the study of cephalometric aspects (lateral and postero-anterior); (iv) the study of the upper airway by an otorhinolaryngologist, such as clinical sinusitis, and/or cysts of the maxillary sinus; (v) the study of odontogenic cysts.

The selected patients were asymptomatic for TMJ’s pain and dysfunction at their first visit. Subjects with unilateral cross bite and with loss of teeth in the posterior zones were excluded (**exclusionary criteria**).

In order to verify whether condylar volume and surfaces might be more characteristic of some types of mandibular divergence angle of facial morphology, the sample was divided on the base of their skeletal class and their mandibular plane angle. The skeletal class was defined on the base of ANB angle, while the mandibular plane angle determined the vertical dimension (GoGn-SN angle).

Subjects were considered in skeletal class I if the ANB angle ranged between 2° ± 2°, and within normal divergence if GoGn-SN angle ranged between 28° and 37°. These measurements were obtained on CBCT using Dolphin software ™.

Subjects in skeletal class I showed a ANB angle of 2 ± 1.5 degree; subjects in skeletal class II showed a ANB angle of 5.5 ± 1.5; subjects in skeletal class III showed a ANB angle of −0.9 ± 0.8 degree.

Subjects in normo-divergence showed a GoGn-SN angle of 33 ± 3 degree; subjects in hyper-divergence showed a GoGn-SN angle of 40 ± 3 degree; subjects in hypo-divergence showed a GoGn-SN of 24 ± 3.

The evaluation of CBCT data included condylar volume and condylar surface.

### The 3D reconstruction

Cone Beam Volumetric Tomography datasets were acquired with the ILUMA™ (IMTEC, 3 M Company, Ardmore, Oklahoma, USA), with a reconstructed layer thickness of 0.5 mm, with a 512x512 matrix. The device was operated at 120 kVp and 3–8 mA by using a high frequency generator with a fixed anode and a 0.5 mm focal spot. A single 40-second high-resolution scan was made of each skull. The voxel size was set at 0.25-mm.

**Figure 1:**
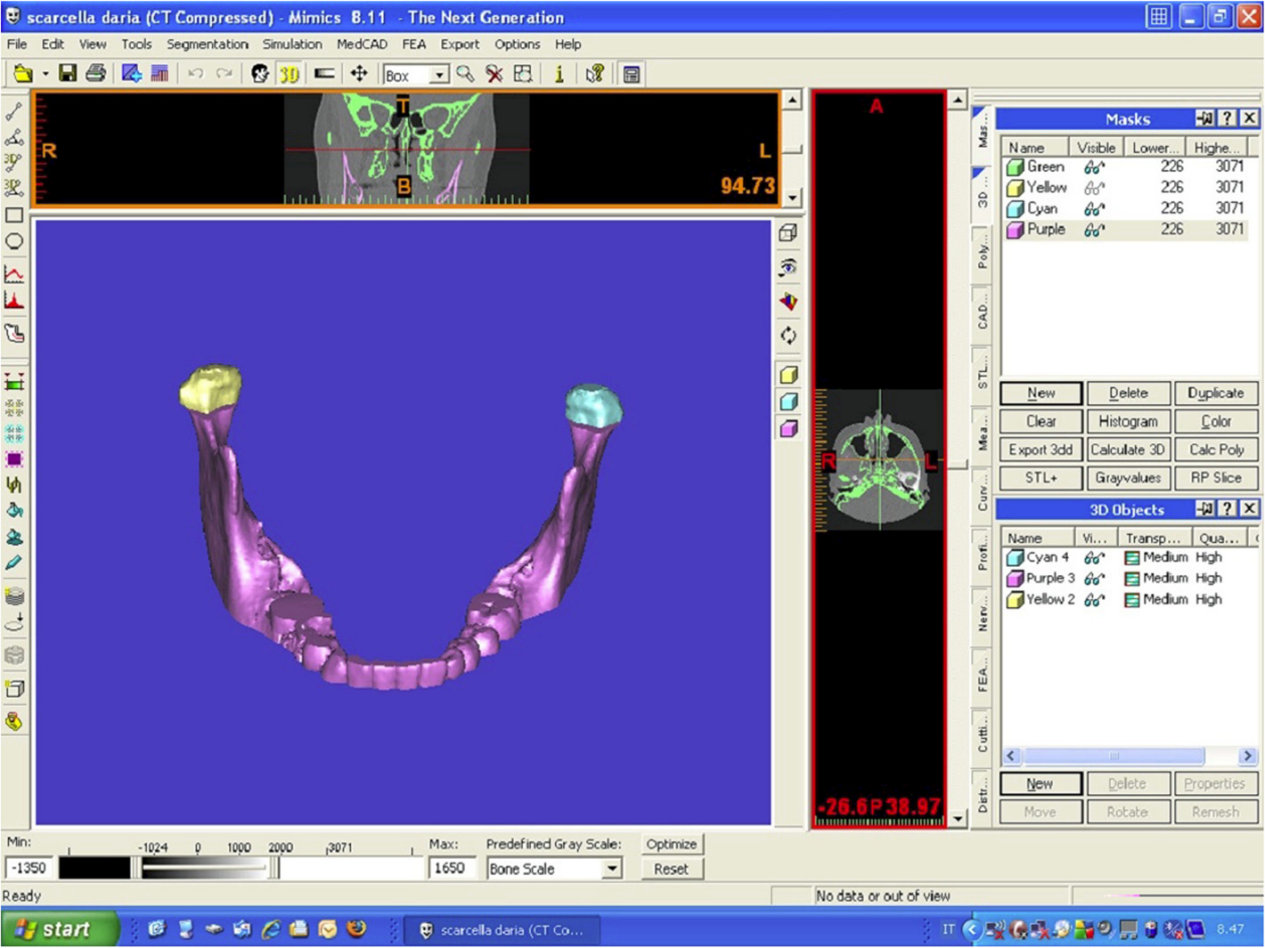
**Condylar 3-D reconstructions with Mimics software.** A mask which includes all mandibular bone structures is created with the Mimics software; the proper threshold for the grey scale is selected to identify the condylar area (1350–1650, in the line under the figure). The black boxes in the upper part of figures and on its right identify the exact position of the two condylar heads on the horizontal plane to define: (i) their antero-posterior distances respect to the frontal plane, and (ii) their right-left distances respect to the sagittal plane.

The segmentation of the mandibular condyle was based on 2D Digital Imaging and Communications in Medicine (DICOM), created with CT data set, using the Mimics™ software 9.0 (Materialise NV Technologielaan, Leuven, Belgium) (Figure 1).

Each condyle was visualized in the recommended bone density range (range of gray scale from −1350 to 1650) and then graphically isolated prior to the 3D and volumetric measurements. Frankfort horizontal (FH) plane was constructed by creating a plane from the inferior orbital rim to the superior border of the external auditory meatus. An initial slice was made parallel to the FH plane just above the superior aspect of the condyle [2].

Then, the area of TMJ was graphically enlarged, and the remaining surrounding structures were progressively removed using various graphical sculpting tools for the upper, the lower and the side condylar walls [11].

**Figure 2:**
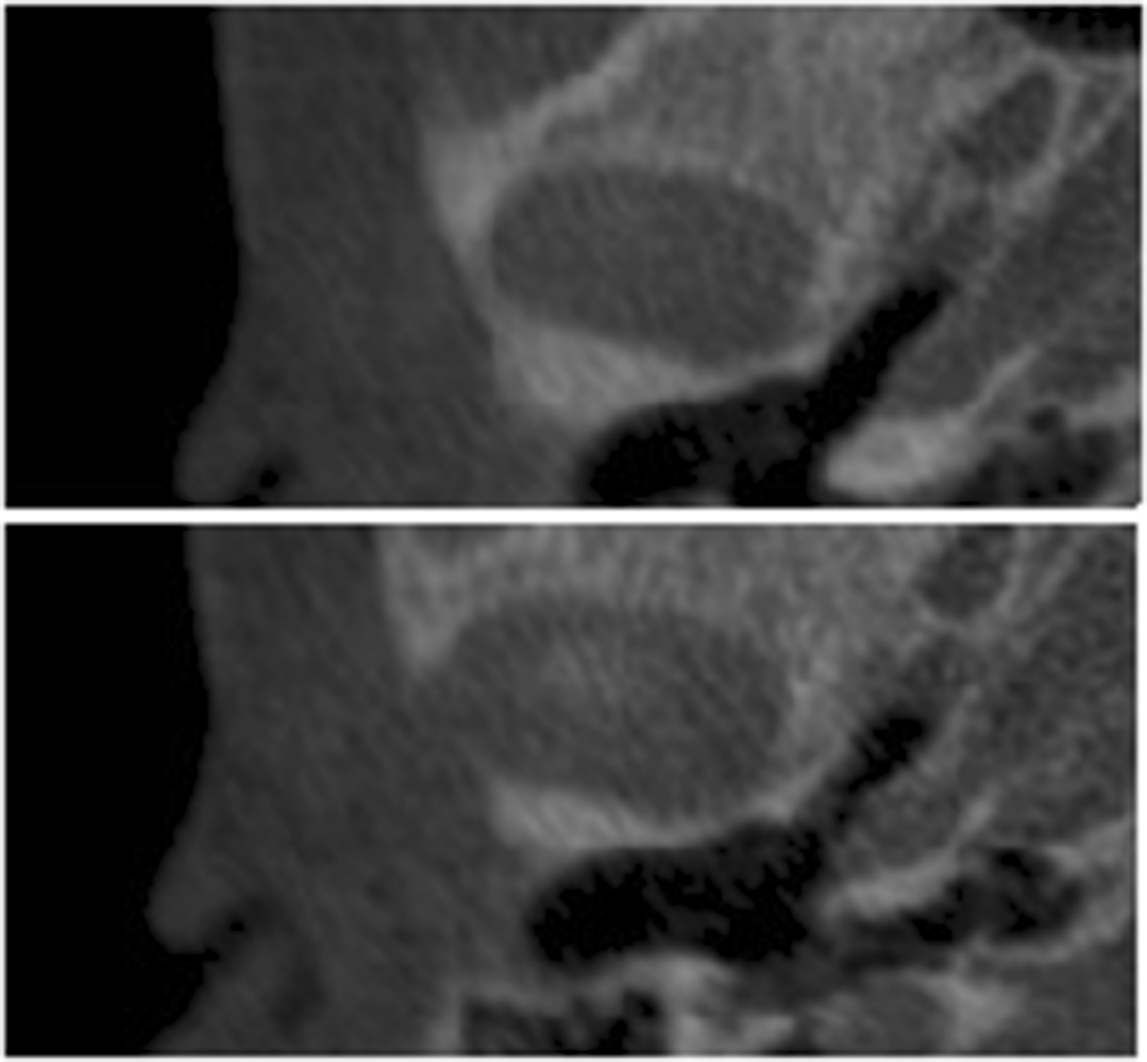
The superior limit of condylar head is selected when the first white area appears in the upper articular region, while scrolling the images from the upper to the lower regions of the joint space.

**Figure 3:**
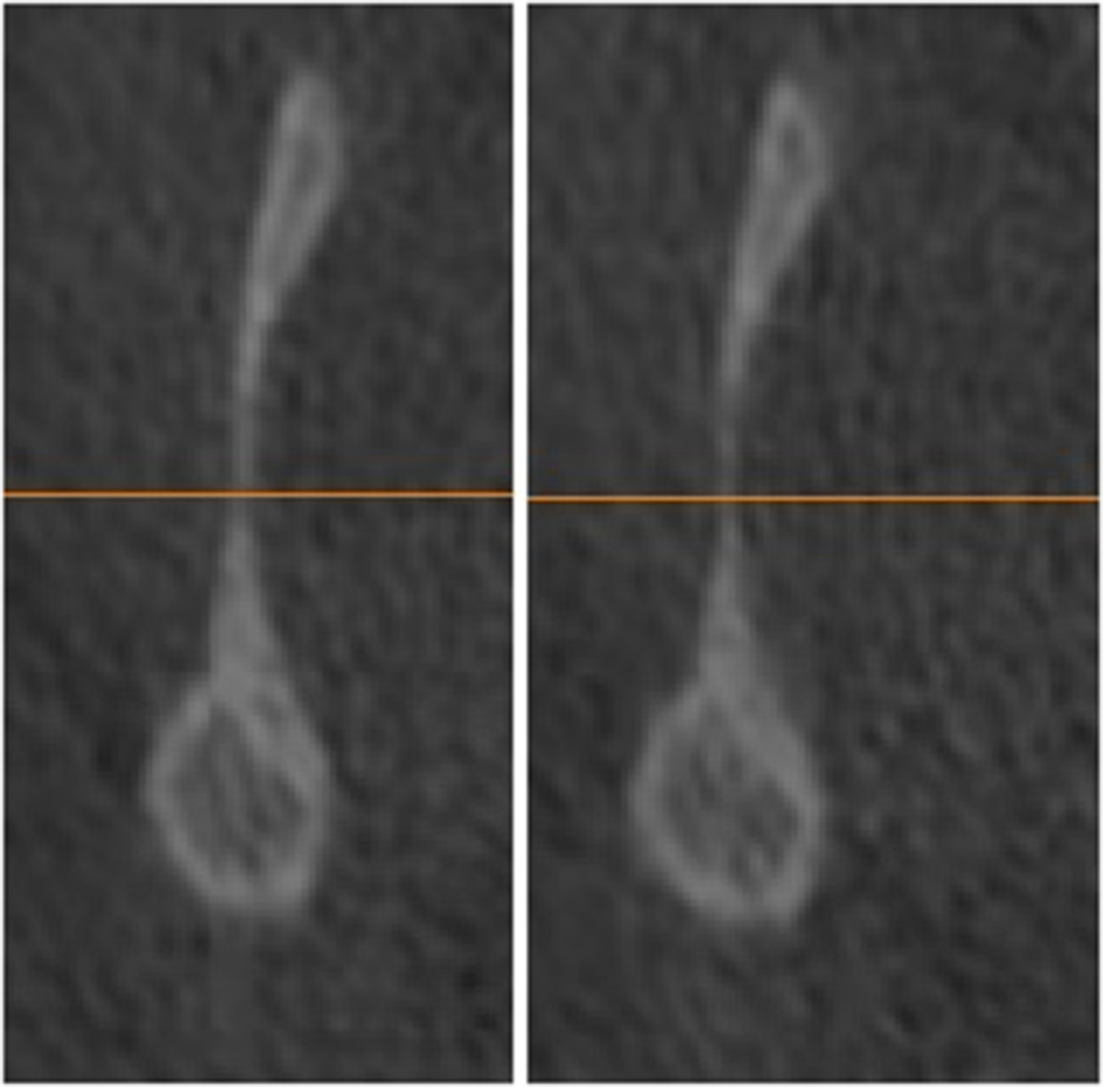
The inferior limit is selected when the sigmoid area disappears.

The upper limit of the condyle was defined where the first radiopaque area was viewed in the area of synovia (Figure 2); then, for each of the lower sections, the condyle was isolated through the visualization of cortical bone. The lower limit of condyle was traced when the section left the ellipsoidal shape (due to the presence of the anterior crest) and became circular suggesting the level of the condylar neck (Figure 3). Once the computer isolations were made, three-dimensional Multiplan reconstructions were produced for each condyle.

Volumetric measurements were made for each condyle with the Mimics™ automatic function.

### Method error analysis

In order to assess intra-observer error in identifying and pinpointing the condylar structure, ten skulls were processed twice by the same operator (S.T.) (with an interval of one day) and differences in condylar volumes and condylar surfaces were evaluated with Wilcoxon test. No significant difference was observed between the two measurements (p = 0.9 for the volume and p = 0.7 for the surface).

In order to assess inter-observer error, the ten skulls were also processed by another researcher (M.S) and the data were compared by using Mann–Whitney test. No significant difference was observed between the two measurements (p = 0.94 for the volume and p = 0.77 for the surface).

### Data analysis

Data were analyzed using SPSS 14.0 (SPSS Inc, Rainbow Technologies, Chicago, Ill). Differences in surface and volumetric measurements across the three groups were analyzed with ANOVA followed by a post-hoc analysis to test the differences among the sets of means of the groups.

### Estimation of the sample size

We performed a study to evaluate the correct number of subjects in the sample for each group. Firstly, we considered the 3 groups. Then, we assigned a P value of 0.01, and a statistical power of 0.8. Then, we determined a minimal acceptable difference equal to the standard deviation of 0.684 observed in the whole sample, and obtained a minimum of 30 subjects for each group. Considering that high angle subjects were only 17, we assigned a power of 0.7 in that case. When we divided the sample on the base of skeletal class, class III subjects came out to be 25 subjects, and we obtained a power of 0.8 in that case.

### Differences between the right and the left sides

Considering the data in the whole sample, we performed a comparison between the data obtained in the right side and the data obtained in the left with the paired sample *T*-test.

No significant difference emerged.

### Differences among the groups

When we compared the three groups with different skeletal classes and the three groups with different mandibular divergence, we tested for differences in volumetric and surface measurements among the three groups with the ANOVA test followed by a post-hoc analysis to assess the significance level.

## Results

Surface graphical rendering revealed detailed images of the left and right condyles based on the processed and registered CBCT data. Visual inspection of the volume images in a 360-degree rotation showed a regular variety of condylar morphology, without any abnormal aspect of condylar anatomy.

Statistical outputs for the whole sample are reported in Tables 1 and 2. No significant difference was found between the right and the left sides for condylar volume and surface. Condylar volume and surface resulted slightly higher in the left side, with respect to the right side, but this small difference was not statistically significant or clinically relevant.

**Table 1:** **Descriptive statistics of condylar volume (mm**^**3**^**) in the whole sample, and in subjects classified on the base of mandibular divergence, or skeletal class**

**Data in the whole sample**						
	N = 34 (15 F and 19 M)	Mean	SD	Minimum	Maximum	P
Volume (right side)	94 (46 F and 48 M)	2535.2	684.7	1058.7	3775.6	NS
Volume (left side)	94(46 F and 48 M)	2569.1	673.2	1006.4	4153.2	
**Low angle group (GoGn-SN angle** **≤** **27°)**						
	N = 34 (15 F and 19 M)	Mean	SD	Minimum	Maximum	P
Volume (right side)	34	2968.08	690.4	1999.1	3775.6	** P < 0.01 versus the other two groups
Volume (left side)	34	3037.4	777.9	2003.3	4153.2	** P < 0.01 versus the other two groups
**Normal angle group (28°** **≤** **GoGn-SN angle** **≤** **37°)**						
	N = 42 (22 F and 20 M)	Mean	SD	Minimum	Maximum	P
Volume (right side)	42	2492.3	709.7	1301.0	3752.2	** P ≤ 0.01 versus the low angle group
Volume (left side)	42	2669.7	742.7	1349.3	3739.3	** P ≤ 0.01 versus the low angle group
**High angle group (GoGn-SN angle** **≥** **37°)**						
	N = 18 (9 F and 9 M)	Mean	Std. Deviation	Minimum	Maximum	P
Volume (right side)	18	2441.2	660.3	1058.7	3608.0	** P ≤ 0.01 versus the high angle group
Volume (left side)	18	2577.5	550.5	1006.4	3183.3	** P ≤ 0.01 versus the high angle group
**Class II group**						
	N = 31 (15 F and 16 M)	Mean	SD	Minimum	Maximum	P
Volume (right side)	31	2520.2	640.5	1058.7	3775.6	NS
Volume (left side)	31	2520.2	640.5	1058.7	3775.6	NS
**Class I group**						
	N = 38 (18 F and 20 M)	Mean	SD	Minimum	Maximum	P
Volume (right side)	38	2580.0	730.2	1495.7	3680.2	NS
Volume (left side)	38	2449.3	780.9	1490.4	3550.3	NS
**Cass III group**						
	N = 25 (13 F and 12 M)	Mean	Std. Deviation	Minimum	Maximum	P
Volume (right side)	25	2592.6	699.6	1006.4	3590.2	NS
Volume (left side)	25	2570.7	679.4	1920.1	3658.3	NS

**Table 2:** **Descriptive statistics of condylar surface (mm**^**2**^**) in the whole sample, and in subjects classified on the base of mandibular divergence, or skeletal class**

**Data in the whole sample**						
		Mean	SD	Minimum	Maximum	P
Surface (right side)	94 (46 F and 48 M)	1230.9	242.7	729.2	1758.1	NS
Surface (left side)	94 (46 F and 48 M)	1249.1	237.3	767.6	1802.9	NS
**Low angle group (GoGn-SN angle** **≤** **27°)**						
	N = 34 (15 F and 19 M)	Mean	SD	Minimum	Maximum	P
Surface (right side)	34	1346.2	187.0	1099.6	1560.1	** p ≤ 0.01 versus the other two groups
Surface (left side)	34	1413.2	275.5	1102.2	1802.9	** p ≤ 0.01 versus the other two groups
**Normal angle group (28°** **≤** **GoGn-SN angle** **≤** **37°)**						
	N = 42 (22 F and 20 M)	Mean	SD	Minimum	Maximum	P
Surface (right side)	42	1225.0	256.3	838.2	1758.1	** p ≤ 0.01 versus the low angle groups
Surface (left side)	42	1288.7	277.9	925.9	1726.4	** p ≤ 0.01 versus the low angle groups
**High angle group (GoGn-SN angle** **≥** **37°)**						
	N = 18					
	(9 F and 9 M)	Mean	Std. Deviation	Minimum	Maximum	P
Surface (right side)	18	1202.5	250.4	729.2	1656.7	** p ≤ 0.01 versus the low angle groups
Surface (left side)	18	1179.3	178.7	767.6	1512.4	** p ≤ 0.01 versus the low angle groups
**Class II group**						
	N = 31 (15 F and 16 M)	Mean	SD	Minimum	Maximum	P
Surface (right side)	31	1280.3	255.4	729.2	1690.2	NS
Surface (left side)	31	1320.4	293.5	870.3	1720.3	NS
**Class I group**						
	N = 38 (18 F and 20 M)	Mean	SD	Minimum	Maximum	P
Surface (right side)	38	1340.4	218.4	889.7	1758.1	NS
Surface (left side)	38	1326.7	248.3	939.5	1802.9	NS
**Class III group**						
	N = 25 (13 F and 12 M)	Mean	Std. Deviation	Minimum	Maximum	P
Surface (right side)	25	1380.6	236.5	990.6	1782.5	NS
Surface (left side)	25	1420.5	237.1	767.6	1790.6	NS

When the subjects with different mandibular divergence were compared, the ANOVA revealed a significant difference (p < 0.01). Post-hoc analysis revealed significant differences between low and high mandibular plane subjects (*t*-test with Bonferroni correction, p < 0.01) (Tables 1 and 2).

There was also a significant difference between low and normal mandibular plane subjects (Mean difference 476.2 ± 160.1; t: 2.960; p < 0.01), while no significant difference was observed between high and normal mandibular plane subjects (Mean difference of 51.3 ± 20.4; t: 0.255; p > 0.01) (Tables 1 and 2).

No significant difference was found among subjects within the three groups with different skeletal class (Tables 1 and 2).

However, it was observed that Class III subjects tended to show higher volume and surface of condylar head, although this was not statistically significant (Tables 1 and 2).

## Discussion

In this study we only included the data of young adult subjects within a limited age range, specify range exactly (mean age 24.3 ± 6.5 years). The anatomy of the mandibular condyle has already been demonstrated to change from childhood to adulthood within an age range from 8.3 years to 42.8 years in 94 joints of 47 subjects [3].

We also excluded subjects with abnormal condylar morphology, due to the development of TMJ osteoarthritis, such as flattening, erosion, sclerosis, osteophytes, resorption. A check of condylar anatomy was performed before the measurement of condylar volume, to assess the regularity of the condylar morphology.

In this study, we considered a homogeneous population with only Caucasian subjects. So it is not possible to clarify whether the relative robustness vs. gracility of the morphology could depend on racial or ethnic type. This could be clarified in a future study including subjects of different ethnic or racial type.

Condylar growth studies in humans using metallic implants have shown that, during the prepubertal or juvenile growth period, mandibular growth takes place at variable rates. Because of the increased intensity of condylar growth during the pubertal growth spurt, the pubertal growth spurt is generally considered to be the best time for orthodontic and functional treatment in patients with Class II malocclusions [12]‐ [14].

Bjork reported that condylar growth rates can vary from as little as 0.5 mm to as much as five mm per year during this period [12]. While Bjork demonstrated variation in growth intensity from year to year in untreated subjects, he did not specifically relate it to any type of occlusion. The purpose of this study was to determine the mandibular condylar volume and surface in a group of young adult subjects, with different skeletal class and mandibular divergence, clinically asymptomatic for TMJ pain and dysfunction using CBCT.

With this method, the study is the first to demonstrate a significant degree of condylar hyperplasia in low mandibular plane angle subjects, compared to normal and high angle subjects.

Our statistical analysis revealed a difference of about 20% in the surface between low- and high- mandibular plane angle subjects and of about 18% in the volume between low- and normal- mandibular plane angle subjects.

The mean differences were lower than the SD value observed in the whole sample, but they are statistically significant and clinically relevant considering the mean percentage of 18-20% with respect to the other groups.

The hypothesis about the mechanism related to the observed differences concerns the muscular activity of the muscles associated to the stomatognatic function.

Another hypothesis about the “mechanism at work” concerns the differences in the genome among subjects with different facial morphological characteristics; the genetic differences could be also related to the histological structures of the condyle; for example, a previous study has demonstrated that hyperplasia of the mandibular condyle is characterized histologically by the presence of an uninterrupted layer of undifferentiated germinative mesenchyme cells, a layer of hypertrophic cartilage and of islands of chondrocytes in the subchondral trabecular bone [4].

Certain conclusions about the mechanism associated to the observed differences are not possible due to the transversal construction of this research; for this, only hypothesis can be done.

From a clinical perspective, these findings lead us to hypothesize that any predisposition to temporomandibular disorders, which seems to differ in subjects with different mandibular skeletal class or divergence could also be related to the condylar volume. This hypothesis needs to be tested by future studies.

For what concerns the condylar size, most articles report on the size in two-dimensional images of the condyle. In a recent study [5] larger spatial measurements (height and width of condyle) in patients with severe Class II malocclusions (mean age 18.0 yrs) were observed with compared with severe class III patients (mean age 19.2 yrs).

During mastication, different force vectors against the condyle exist [6] and also class II subjects show a larger direction of the force vector compared with class I and class III subjects [7].

There are a few reports suggesting that TMJ morphology has a strong correlation with skeletal morphology [7] and an exclusively inverse relationship between the angle of the articular eminence and the occlusal and the mandibular planes in asymmetric subjects, since a small angle of eminence to FH plane and the large superior condylar space were observed in the asymmetric skeletal III subjects [8].

In the literature, the condylar volume has been also related to the type of mastication [9]. In a study with twenty-five subjects submitted to hard diets, soft diets, or alternate hard and soft diets, the condylar width was significantly greater in the hard diet group than in the soft diet group after one week, suggesting that changes in mastication markedly affect mandibular condylar cartilage growth and mandibular morphology.

Throughout this study, however, the observed differences were in the range of the standard deviation observed in the whole sample, and the extent to which such differences can be considered physiological or pathological still remains unclear even though there are significant differences between groups, with a difference of about 18-20%. Quantitative information on condylar volume can not tell anything about TMJ signs and symptoms or any particular morphological alteration, as we included only asymptomatic subjects with malocclusion, so the observed variability seems to complicate correct diagnosis and treatment planning.

Owing to ethical reasons, it was not possible to include other subjects (we only performed a retrospective evaluation) or to schedule another CBCT procedure over time. For both reasons, the interpretation of the results remains difficult.

Our pilot evaluation of the volume images in 360 degree represents an initial classification, which should be supported by new studies.

## Conclusion

In the present study, using the CBCT-based method, it was shown that the condylar volume and surface could be measured accurately. In a group of subjects with different mandibular angles and skeletal classes, condylar volume was found to be a common feature. The degree of difference among the various groups was variable, but significant in the low mandibular angle subjects compared to normal and high angle subjects.

Condylar size, both volume and surface, seems to correlate with the mandibular morphology, therefore influencing facial divergence and, at a lower rate, skeletal class of a subject, mostly in the low mandibular angle subjects.

## Competing interests

The authors declare that they have no competing interests.

## Authors’ contributions

MS and ST are the Principal Investigator of this research article. They 1) have made substantial contributions to conception and design, acquisition of data, analysis and interpretation of data; 2) have been involved in drafting the manuscript or revising it critically for important intellectual content; and 3) have given final approval of the version to be published. AP and FF have made substantial contributions in acquisition of data and participated in drafting the manuscript and helped in the revision of the manuscript. All authors read and approved the final manuscript.
